# A critical role for CaMKII in behavioral timescale synaptic plasticity in hippocampal CA1 pyramidal neurons

**DOI:** 10.1126/sciadv.adi3088

**Published:** 2023-09-06

**Authors:** Kuo Xiao, Yiding Li, Raymond A. Chitwood, Jeffrey C. Magee

**Affiliations:** ^1^ Department of Neuroscience, Baylor College of Medicine, Houston, TX, USA.; ^2^ Howard Hughes Medical Institute, Baylor College of Medicine, Houston, TX, USA.

## Abstract

Behavioral timescale synaptic plasticity (BTSP) is a type of non-Hebbian synaptic plasticity reported to underlie place field formation. Despite this important function, the molecular mechanisms underlying BTSP are poorly understood. The α-calcium-calmodulin-dependent protein kinase II (αCaMKII) is activated by synaptic transmission–mediated calcium influx, and its subsequent phosphorylation is central to synaptic plasticity. Because the activity of αCaMKII is known to outlast the event triggering phosphorylation, we hypothesized that it could mediate the extended timescale of BTSP. To examine the role of αCaMKII in BTSP, we performed whole-cell in vivo and in vitro recordings in CA1 pyramidal neurons from mice engineered with a point mutation at the autophosphorylation site (T286A) causing accelerated signaling kinetics. Here, we demonstrate a profound deficit in synaptic plasticity, strongly suggesting that αCaMKII signaling is required for BTSP. This study elucidates part of the molecular mechanism of BTSP and provides insight into the function of αCaMKII in place cell formation and ultimately learning and memory.

## Teaser

The molecular mechanisms of BTSP have been revealed to require the autophosphorylation of CaMKII.

## INTRODUCTION

The hippocampus is important for spatial memory in humans and rodents (*1*–*5*). During exploratory behavior, hippocampal pyramidal neurons have been observed to fire action potentials (APs) at specific locations in the environment (*1*, *6*, *7*). These “place cells” (PCs) are believed to be formed by behavioral timescale synaptic plasticity (BTSP), where the weights of active synaptic inputs are bidirectionally altered by a single initiation event—a global voltage signal termed a plateau potential—temporally separated from the to-be-modified synaptic input by several seconds (*8*). BTSP has been observed to create new place fields in multiple experimental paradigms including in vivo imaging and whole-cell patch recordings, juxtacellular stimulation, and optogenetic activation of pyramidal neurons in behaving mice (*8*–*15*). BTSP has also been demonstrated in CA1 pyramidal neurons in vitro (*8*, *16*). These results suggest that BTSP is a robust synaptic plasticity mechanism for rapidly producing PCs in the hippocampus.

**Fig. 1.**
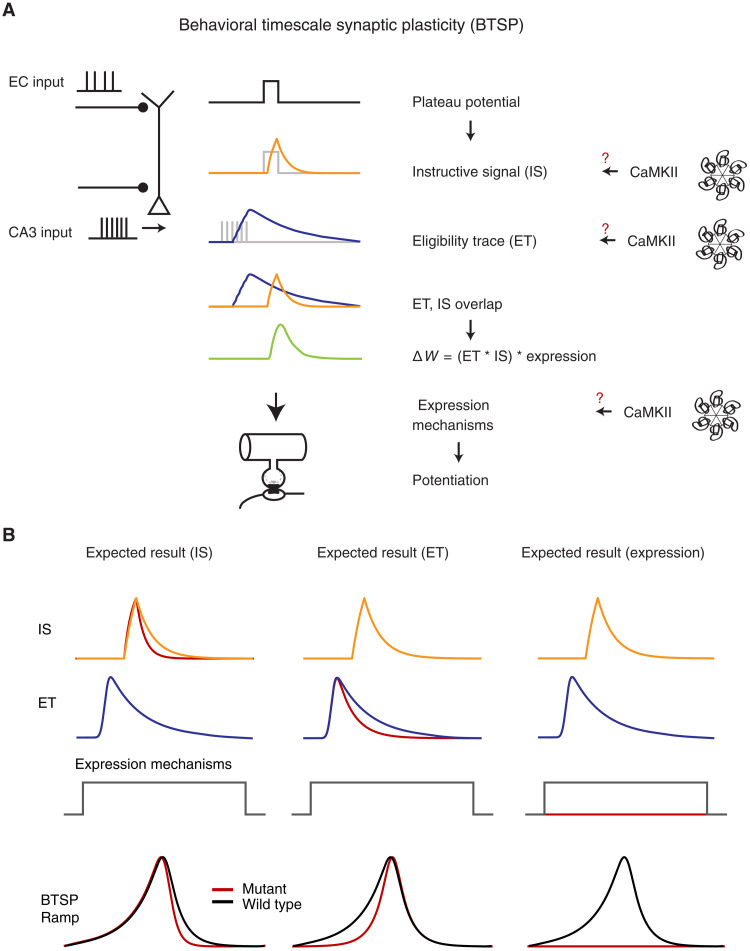
Overview of BTSP and the potential involvement of CaMKII. (**A**) BTSP model summary. The BTSP model consists of two primary components: an eligibility trace (ET) and an instructive signal (IS). ETs are induced by CA3 inputs, while the IS arises from plateau potentials initiated in the tuft region. Gray traces represent electrical events, which produce filtered biochemical signals as shown as colored traces in the schematic. The interaction between ETs and IS determines the delta weight (Δ*W*) amplitude. We hypothesize that CaMKII plays a role in BTSP, potentially underlying the IS, ET, or expression mechanism (red question marks). (**B**) Anticipated experimental outcomes. CaMKII could be implicated in the IS, ETs, or an expression mechanism. Depending on its involvement, distinct effects on the BTSP ramp (depolarization of *V*_m_ due to BTSP) would be observed: a more asymmetric ramp shape if CaMKII affects the IS, a more symmetric ramp shape if it influences ETs, and the disappearance of the BTSP ramp if CaMKII is involved in the expression mechanism.

BTSP has been shown to require *N*-methyl-d-aspartate receptor and l-type Ca^2+^ channel activity, but, other than this, the molecular mechanisms involved in BTSP are relatively unknown (*8*). While it seems reasonable to expect that BTSP will share many expression mechanisms with standard LTP, the long time course of BTSP suggests the necessity of two additional signals. That is, additional molecular mechanisms are needed to temporally filter both the plateau potential [an instructive signal (IS)] and synaptic input [an eligibility trace (ET)] (Fig. 1A). Although the concept of molecular activity filters (ETs) is nearly 50 years old, there is scant evidence concerning the molecules potentially involved (*17*–*21*).

Several lines of evidence suggest that α-Ca^2+^-calmodulin-dependent protein kinase II (αCaMKII) plays a central role in multiple forms of synaptic plasticity, and various properties of αCaMKII identify it as a potential molecular candidate of either the IS or the ET (*22*–*25*). The multimeric structure of the αCaMKII holoenzyme endows this molecule with the ability to maintain a phosphorylated state for an extended period [decay time constant of 8.2 s; (*26*)] following transient increases in Ca^2+^ (*26*). At the molecular level, this is mediated by the binding of Ca^2+^/calmodulin (Ca^2+^-CaM) to adjacent regulatory subunits, resulting in the phosphorlylation of T286 in the autoinhibitory portion of the regulatory domain. This disinhibition allows for the kinase activity to persist beyond the initial binding of Ca^2+^-CaM (*27*). Therefore, T286 autophosphorylation allows for the integration of transient Ca^2+^ signals and their transformation into long-lasting αCaMKII activation (*28*, *29*). The substitution of Thr^286^ (T) for alanine (A; T286A) of αCaMKII has been demonstrated to prevent CaMKII constitutive activity, and animals engineered to express this mutation perform poorly in spatial memory tasks and display deficits in hippocampal synaptic plasticity and reductions in spatial selectivity and stability of PCs (*23*, *24*). Recent studies using Förster resonance energy transfer–based CaMKII sensors revealed that this mutation shortens the duration of αCaMKII activation several fold [decay time constant of 1.9 s; (*26*)] relative to the native autophosphorylated state (*26*, *27*).

On the basis of the above studies, we hypothesize that αCaMKII activity is involved in BTSP. If αCaMKII activity functions as either an ET or an IS, then the more rapid decay kinetics resulting from the T286A mutation should decrease the duration of these signals (Fig. 1B). This reduced time course would significantly alter the shape of the underlying subthreshold membrane potential (*V*_m_ ramp) produced by the BTSP potentiated synapses with the exact alteration dependent on which signal (IS or ET) is mediated by αCaMKII. Alternatively, αCaMKII signaling might be a component of the myriad synaptic plasticity expression mechanisms, and, in this case, the modified kinetics resulting from the T286A mutation would affect the level of BTSP induction perhaps to the point where BTSP is unable to produce PCs or even any associated subthreshold *V*_m_ changes (Fig. 1B). Last, if αCaMKII signaling is not involved in BTSP, then the T286A mutation would have no effect. Thus, the T286A αCaMKII mutation can aid in teasing apart the specific role of this molecule in BTSP.

To test the effect of faster αCaMKII decay kinetics on BTSP, we performed whole-cell patch-clamp recordings in awake behaving *T286A^+/+^* (Homo) mice, as well as *T286A^+/−^* (Het) and wild-type (WT) mice as controls. We found that the BTSP induction in the *T286A^+/+^* group resulted in very weak or no BTSP, whereas the Het group and the WT group have similar BTSP. We also observed an increased propensity for CA1 neurons in the *T286A^+/+^* group to fire spontaneous plateau potentials, suggesting alterations in excitability in either CA1 or other regions of the hippocampal network. These spontaneous plateaus in the homozygous group also did not produce PC activity. To control for the possibility that the above effect of T286A mutation resulted from an alteration in the hippocampal network such that CA1 received only inconsistent afferent input, we tested BTSP induction in hippocampal brain slice, where we can reliably and consistently activate a given set of synaptic inputs. Consistent with the in vivo results, BTSP induction here produced minimal potentiation in the T286A slices relative to control. In addition, standard in vitro measures of membrane excitability did not reveal differences that would likely contribute to the increase in spontaneous plateau potentials observed during behavior. Together, our results reveal that αCaMKII plays a central role in BTSP at some stage other than in the generation of ETs or ISs, suggesting that αCaMKII signaling is likely required for the synaptic plasticity expression process.

## RESULTS

### Experimental design and behavioral quantification

**Fig. 2.**
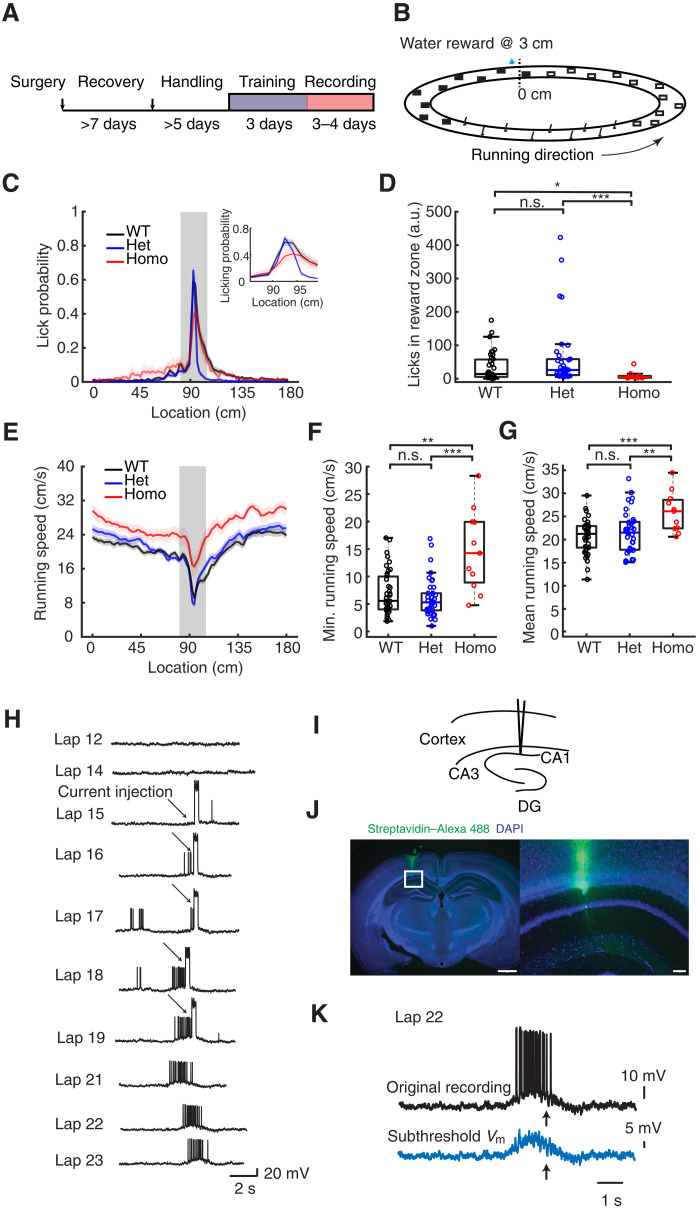
Experimental setup, behavioral quantification, and example BTSP recordings. (**A**) Experimental timeline. (**B**) Linear track configuration with cues; reward site at 3 cm. (**C**) Licking probability across spatial locations for WT, Het, and Homo groups. Insets display an expansion of the reward site region. Heavy lines are averages of all laps (reward at 3 cm). Shading represents SEM. (**D**) Relative licking probability increase in the reward zone (see Materials and Methods). *H* = 14.88, *P* = 5.87 × 10^−4^ for the Kruskal-Wallis test. *Z* = −1.94 (WT versus Het), 2.32 (WT versus Homo), and 3.87 (Het versus Homo). *P* = 0.131 (WT versus Het), 0.0414 (WT versus Homo), and 4.71 × 10^−4^ (Het versus Homo). a.u., arbitrary units. (**E**) Running speed across spatial locations. (**F**) Minimum running speed for all laps. *H* = 15.0, *P* = 5.53 × 10^−4^ for the Kruskal-Wallis test. *Z* = 0.871 (WT versus Het), −3.32 (WT versus Homo), and −3.75 (Het versus Homo). *P* = 0.774 (WT versus Het), 0.00305 (WT versus Homo), and 3.58e × 10^−4^ (Het versus Homo). (**G**) Mean running speed. *F* = 7.28, *P* = 0.0012 for the analysis of variance (ANOVA). *T* = −0.993 (WT versus Het), −4.10 (WT versus Homo), and −2.91 (Het versus Homo). *P* = 0.979 (WT versus Het), 8.00 × 10^−4^ (WT versus Homo), and 0.00705 (Het versus Homo). (**H**) Example WT cell following BTSP induction, producing a field in a previously silent CA1 neuron. Arrows indicate current injections. Scale, 20 mV and 2 s. (**I**) Schematic of recording site. (**J**) Example histology of a CA1 neuron. Blue, 4′,6-diamidino-2-phenylindole (DAPI); green, streptavidin 488. Scale bars, 1000 μm (left) and 100 μm (right). (**K**) Process for subthreshold place field voltage calculation as described previously (*8*). Parametric analysis used one-way ANOVA with Bonferroni correction; nonparametric analysis used the Kruskal-Wallis test with Dunn’s test for multiple comparisons. *P < 0.05, **P < 0.01, and ***P < 0.001; n.s., not significant (*P* ≥ 0.05). Sample sizes: *n* = 39 (WT) from 24 mice, *n* = 38 (Het) from 26 mice, and *n* = 11 (Homo) from 7 mice.

To determine the effect of altered αCaMKII signaling on PC formation, we performed whole-cell recordings from CA1 pyramidal neurons in awake behaving mice. Animals were well acclimated to the experimenter and trained to run on a treadmill to receive a water reward at a fixed location (Fig. 2A). The treadmill was a 180-cm belt demarked with distinct visual and tactile cues on its surface (Fig. 2B). As described previously (*30*), well-trained animals exhibited stereotyped patterns of running and licking as they neared the reward location (Fig. 2, C to G). The fraction of licks within a 15-cm zone centered around the reward location were similar in the WT and Het groups but significantly reduced in the Homo group (Fig. 2, C and D). The homozygous animals typically displayed faster running speed across the track, and the minimum speed at the reward site was faster compared to the WT and Het groups (Fig. 2, E to G). These differences are suggestive of spatial learning deficits in the Homo group, as described previously (*24*, *31*). Despite these quantitative differences, the characteristic increases in licking and slowing near the reward zone indicated that the homozygous animals engaged in the task and could be directly compared to the WT and Het groups.

As described previously, BTSP can be reliably induced by strong somatic depolarization that evokes plateau potentials to establish location-specific firing (*10*, *15*, *32*, *33*). In WT animals, CA1 pyramidal neurons are typically silent, but brief large-amplitude current injections (300 ms, ~700 pA) at a defined location will transform silent cells into PCs. Although our standard induction protocol uses five repetitions of somatic current injection, the location-specific firing of APs often develops after the first induction trial and grows more pronounced with subsequent trials (Fig. 2, H to J). The AP output of this newly formed PC begins at a location preceding location of induction presumably because synaptic inputs active at these locations have been potentiated (*8*). When the membrane potential is filtered to include only subthreshold values, the underlying *V*_m_ ramp begins at locations preceding the location of induction (Fig. 2K) and is prospectively asymmetric.

### *V*_m_ ramp

**Fig. 3.**
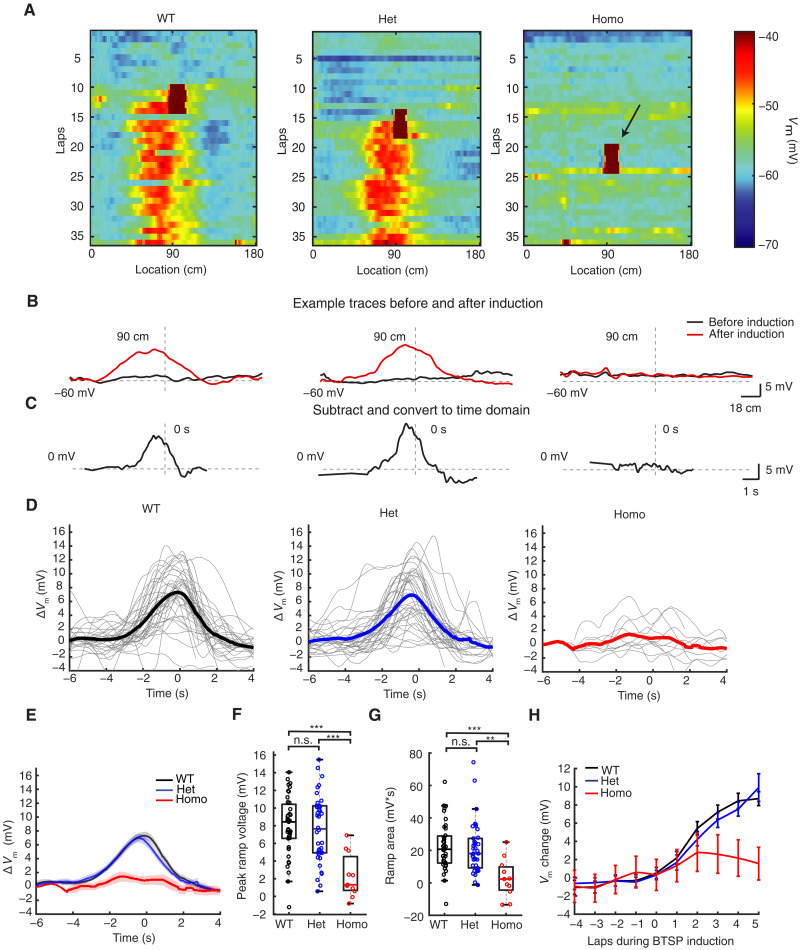
Reduced BTSP in vivo in CaMKII T286A homozygous mutant mice. (**A**) Color plot of three example cells from the WT, Het, and Homo groups, displaying subthreshold membrane potential for each lap. Arrows indicate plateau potentials during BTSP induction events. (**B** and **C**) Example traces showing the ramp after BTSP induction for the WT, Het, and Homo groups. The subthreshold *V*_m_ before and after BTSP induction was averaged (five laps before and five laps after), and the resulting ramp is obtained by subtracting the preinduction trace from the postinduction trace. The subtraction trace is converted into time domain based on the fastest running speed during induction laps, as described by (*8*). Dashed lines indicate −60 and 0 mV, 90-cm location, and 0 s. (**D**) BTSP ramps for each cell in the WT (left), Het (middle), and Homo (right) groups; thicker lines represent group averages. (**E**) Averaged data for all groups, with shading indicating means ± SEM. (**F**) Quantification of ramp peak. *F* = 12.0, *P* = 2.48 × 10^−5^ for the ANOVA test. *T* = 0.445 (WT versus Het), −5.15 (WT versus Homo), and 4.41 (Het versus Homo). *P* = 1.00 (WT versus Het), 2.35 × 10^−5^ (WT versus Homo), and 7.69 × 10^−5^ (Het versus Homo). (**G**) Quantification of the ramp area. *H* = 14.9, *P* = 5.85 × 10^−4^ for the Kruskal-Wallis test. *Z* = 0.769 (WT versus Het), 3.65 (WT versus Homo), and 3.44 (Het versus Homo). *P* = 0.854 (WT versus Het), 4.07 × 10^−4^ (WT versus Homo), and 0.00262 (Het versus Homo). (**H**) Speed of BTSP ramp formation, depicting subthreshold membrane potential in the place before (5.4 cm before) the plateau induction for different laps (five laps before induction and five laps during induction). One-way ANOVA with Bonferroni correction. **P < 0.01 and ***P < 0.001; n.s. (*P* ≥ 0.05). Sample sizes: *n* = 39 (WT) from 24 mice, *n* = 38 (Het) from 26 mice, and *n* = 11 (Homo) from 7 mice.

Because αCaMKII signaling acts to temporally filter the postsynaptic changes in *V*_m_ and associated Ca^2+^ influx in a manner consistent with the molecular underpinning of either an ET or an IS and the T286A mutation is known to hasten the decay of this signaling and adversely affect synaptic plasticity, PC stability, and spatial memory, we compared the *V*_m_ ramp resulting from a standard BTSP induction protocol in all groups of animals. In the WT and Het groups, comparisons of the subthreshold *V*_m_ before and after BTSP induction revealed the instantaneous formation of depolarizing synaptic responses for many seconds around the induction location (90 cm). However, little or no such changes in subthreshold *V*_m_ were observed in the homozygous group (Fig. 3A). A further comparison of the average subthreshold *V*_m_ for the five laps before and after BTSP induction reveals a characteristic asymmetric *V*_m_ ramp for the WT and Het groups, which peaked at 8.0 ± 0.54 mV and 7.6 ± 0.62 mV, respectively. In contrast, the subthreshold *V*_m_ in the homozygous group was minimally increased by 2.3 ± 0.75 mV following BTSP induction (Fig. 3, B to G). The evolution of the *V*_m_ ramp during the induction protocol was similar for the WT and Het groups, exceeding 8 mV within five laps after the start of BTSP induction, while the change in *V*_m_ in the Homo group was <2 mV (Fig. 3H). To control for the possibility that somatic depolarization might be ineffective at reproducing the conditions of a naturally occurring plateau potential in the homozygous group, we also compared the subthreshold *V*_m_ before and after spontaneous plateau events and found that these were also ineffective at producing *V*_m_ ramps in these mice (fig. S1).

### Slice experiments

**Fig. 4.**
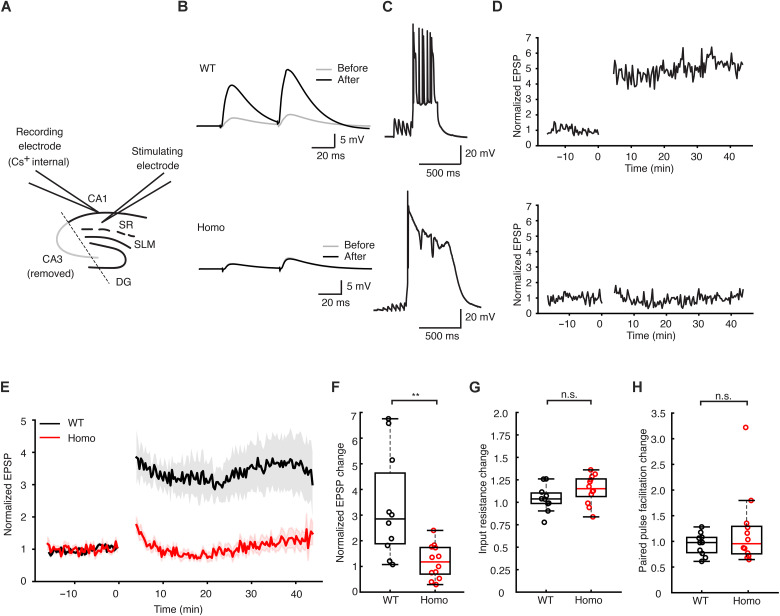
Reduced BTSP in CaMKII T286A homozygous mutant mice in slice experiments. (**A**) Schematic of the slice experiment, with excitatory postsynaptic potentials (EPSPs) elicited by electrical stimulation in the *stratum radiatum* (SR) area of the hippocampus; SLM, *stratum lacunosum-moleculare*; DG, dentate gyrus. CA3 region was removed to prevent excessive input from CA3 neurons interfering with recordings. (**B**) Example raw traces of EPSPs before and after BTSP induction in the WT group (top) and in the Homo group (bottom). (**C**) Example traces during the BTSP inductions from the WT group (top) and the Homo group (bottom). (**D**) EPSP amplitude plotted against time, with cells recorded for over 15 min before induction to ensure stable baselines. Postinduction recordings last up to 40 min. (**E**) Group data for EPSP amplitude plotted against time, with lines and shaded backgrounds representing means ± SEM, respectively. (**F**) Quantification of the EPSP amplitude change. The change is calculated by dividing the averaged EPSP amplitude before induction from the EPSP amplitude after induction in each of the cells. *T* = 3.39, *P* = 0.00291. (**G**) Quantification of the changed input resistance (before and after induction) of the WT and homozygous groups. *T* = −1.52, *P* = 0.144. (**H**) Quantification of the changed paired-pulse facilitation rate (before and after induction) of the WT and homozygous groups. The paired-pulse facilitation rate was calculated by dividing the first EPSP’s amplitude from the second EPSP’s amplitude. *Z* = −0.528, *P* = 0.598 (rank sum test). Statistical analyses used two-sample Student’s *t* test for parametric tests and Wilcoxon rank sum test for nonparametric test. **P < 0.01; n.s. (*P* ≥ 0.05). Sample sizes: *n* = 10 (WT) from eight mice and *n* = 12 (Homo) from five mice.

The plasticity observed following BTSP induction is believed to be the result of increases in synaptic weight of spatially tuned inputs reliably activated at specific locations as the animal traverses the environment. Because the effect of the T286A mutation may have produced alterations to the circuit providing these inputs, it is possible that the reliability of these inputs being consistently active at a given location may be diminished to the point where it would be unlikely to observe plasticity. To control for this, we next attempted to induce BTSP in the slice preparation where the population of synaptic inputs activated by extracellular stimulation would be more consistent on a trial-to-trial basis (Fig. 4A). To reliably elicit plateau potentials, we used a Cs^+^-based internal solution as described previously (*8*). Consistent with previous reports, BTSP induction at the 0-ms interval produced an immediate 3.34- ± 0.63-fold increase in synaptic weight relative to baseline in slices from WT animals. In contrast, slices from the homozygous animals exhibited very weak BTSP with a modest 1.19- ± 0.18-fold increase relative to baseline (Fig. 4, B to F, and fig. S2). BTSP induction did not produce changes in input resistance or paired-pulse facilitation relative to baseline in either group (Fig. 4, G and H). These results add further support to the in vivo findings, suggesting a critical role for αCaMKII in BTSP that is likely not related to the filtering of either the synaptic input (i.e., ETs) or the plateau potential (i.e., ISs).

### Cellular excitability

**Fig. 5.**
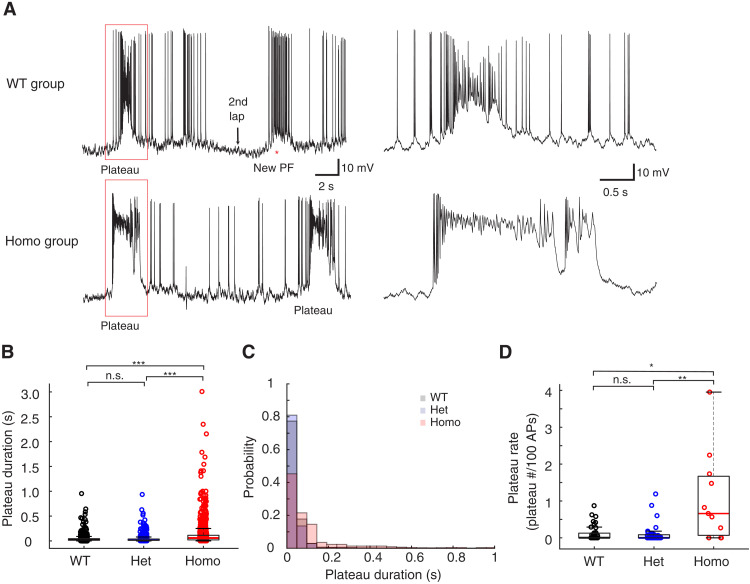
Cellular excitability comparison between WT and CaMKII T286A mutant neurons from in vivo recordings. (**A**) Example traces of the WT group (top) and the Homo group (bottom). (**B**) Quantification of the plateau potential duration in three groups. All plateau potential events with a peak voltage that is more depolarized than −35 mV are used for analysis. Permutation test, Hedge’s *g* value: 0.02 (WT versus Het), −0.60 (WT versus Homo), and −0.52 (Het versus Homo). *P* = 0.254 (WT versus Het), 3.33 × 10^−5^ (WT versus Homo), and 3.33 × 10^−5^ (Het versus Homo); *n* = 1650, 964, and 989 for each group, respectively. (**C**) The distribution of the duration of all the plateau potentials. The Homo group has longer duration plateau potentials. (**D**) Quantification of the plateau potential rate. Plateau rate is defined as the number of plateau potentials occurring for every 100 single (non-plateau) APs as described previously (*32*). Only plateau potentials with more than 50-ms duration were quantified. *H* = 9.79, *P* = 0.0075 for the Kruskal-Wallis test. *Z* = 0.681 (WT versus Het), −2.75 (WT versus Homo), and −2.93 (Het versus Homo). *P* = 0.900 (WT versus Het), 0.0212 (WT versus Homo), and 0.00586 (Het versus Homo). Kruskal-Wallis test with Dunn’s test for multiple comparisons was used in (D). Permutation test with Bonferroni correction was conducted in (B). *P < 0.05, **P < 0.01, and ***P < 0.001; n.s. (*P* ≥ 0.05). Sample sizes: *n* = 39 (WT) from 24 mice, *n* = 38 (Het) from 26 mice, and *n* = 11 (Homo) from 7 mice.

**Fig. 6.**
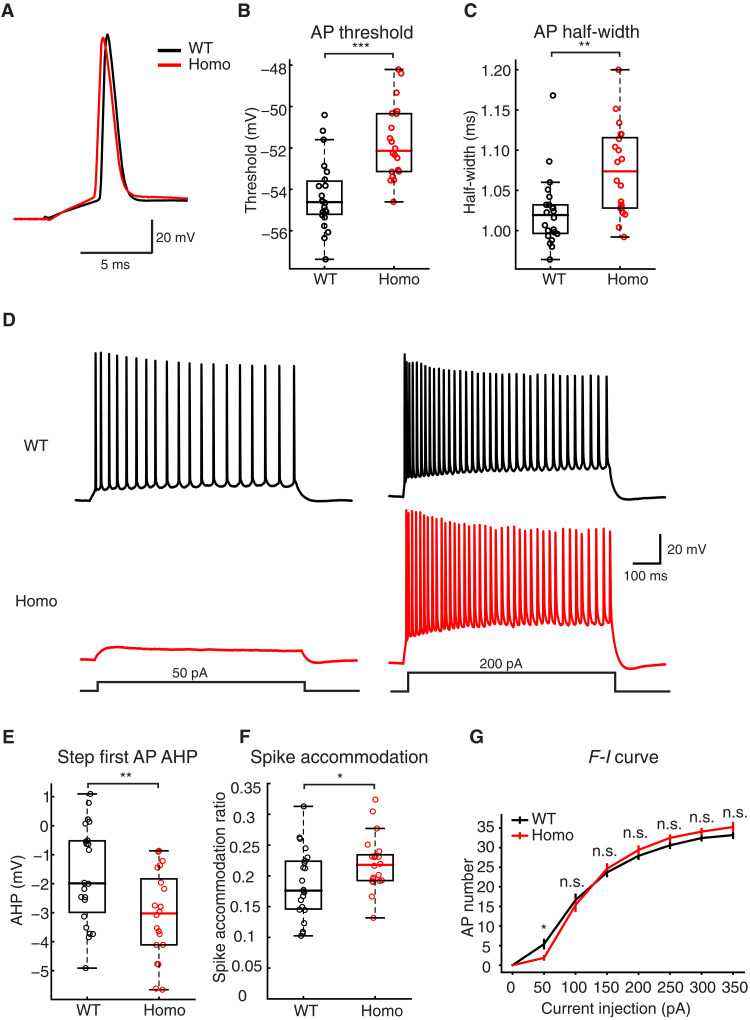
Cellular excitability comparison between WT and CaMKII T286A mutant neurons from in vitro recordings. (**A**) Example trace of single AP recorded from brain slices in the WT and Homo animals. (**B**) Quantification of the AP threshold. *T* = −4.80, *P* = 2.25 × 10^−5^. (**C**) Quantification of the AP half-width. *Z* = −3.15, *P* = 0.00164. (**D**) Example traces recorded from brain slices with step current injection in WT and Homo group mice. Only the 50 and 200 pA traces were shown here for clarity. (**E**) Quantification of the after-hyperpolarization (AHP) in the first AP elicited by step current injection (0 to 350 pA in 50-pA steps). The AHP is calculated by subtracting the threshold from the minimum between the first and second APs. *T* = 2.77, *P* = 0.00854. (**F**) Quantification of spike accommodation in WT and Homo cells with step current injection. Spike accommodation is calculated by dividing the first inter spike interval and the last inter spike interval with 350-pA current injection [see (*37*)]. *T* = −2.15, *P* = 0.0376. (**G**) The comparison of the frequency-current (*F*-*I*) curve in the WT group and the Homo group. *P* = 0.0219 (rank sum), 0.608 (*t* test), 0.627 (*t* test), 0.336 (*t* test), 0.156 (*t* test), 0.215 (*t* test), and 0.0607 (rank sum). Statistics: 2.29 (*Z*), 0.518 (*T*), −0.489 (*T*), −0.973 (*T*), −1.45 (*T*), −1.26 (*T*), and −1.88 (*Z*). To analyze the *F*-*I* curve, every individual cell’s *F*-*I* curve was fitted to a sigmoid function. The midpoint of every fitting result was used for comparison between groups (fig. S3F). Two-sample Student’s *t* test is conducted for all parametric analyses. Mann-Whitney *U* test is conducted for all non-parametric analyses. *P < 0.05, **P < 0.01, and ***P < 0.001; n.s. (*P* ≥ 0.05). Sample sizes: *n* = 22 (WT) from three mice and *n* = 20 (Homo) from three mice.

Independent of BTSP induction, the spiking behavior of CA1 pyramidal neurons in the T286A animals was different with regard to the probability and duration of spontaneous plateau events (Fig. 5A). The duration of these spontaneous plateaus was 3.44- and 3.52-fold longer compared to the WT and Het groups (Fig. 5, B and C). The probability of observing a spontaneous plateau was 8.8-fold greater in the homozygous group relative to that in the WT group and 10.1-fold greater than that in the Het group (Fig. 5D). There were no differences found when comparing input resistance (*R*_N_), AP half-width, or AP firing rates in either the standing or running phases of the behavioral task (fig. S3). Because the recording conditions in the in vivo experiments were suboptimal in terms of access resistance, we repeated a set of experiments in the slice to more accurately compare the electrical properties of CA1 neurons between the WT and homozygous groups. A comparison of single APs resulting from brief depolarizing current injections at the soma revealed a more hyperpolarized AP threshold (WT, −54.3 ± 0.352; Homo, −51.7 ± 0.389 mV; Fig. 6, A and B) and shorter AP duration in the WT group (WT, 1.02 ± 0.009; Homo, 1.07 ± 0.012 ms; Fig. 6C) despite similar values for resting potential, AP amplitude, maximum *dV*/*dt*, *R*_N_, and sag ratio (fig. S3, A to E). In response to longer depolarizing current steps, the AP output as a function of current amplitude was similar for all but the 50-pA step, likely reflecting the difference in AP threshold (Fig. 6, D to G, and fig. S3F).

## DISCUSSION

We have demonstrated a critical role for αCaMKII in hippocampal CA1 BTSP using a combination of in vivo and in vitro experimental platforms. The T286A point mutation markedly reduced the formation of PCs by synaptic plasticity induced by both spontaneous and induced plateau potentials as animals homozygous for this mutation performed a spatial navigation task. In contrast, the same interaction of plateau potentials and active synapses reliably produced PC activity in the WT or Het animals (Figs. 2 and 3). These results suggest that αCaMKII does not function as either an IS produced by plateau potential generation or an ET resulting from appropriate synaptic activation. The fact that the T286A point mutation greatly inhibited the magnitude of the plasticity instead of simply altering the shape of the resulting subthreshold *V*_m_ ramp (Fig. 1) suggests a role for αCaMKII signaling in the expression of BTSP.

The observation of an increased probability of spontaneous plateau potential occurrence in the homozygous T286A mice suggests the possibility of alterations in the hippocampal circuit and/or cellular excitability. If the underlying mechanisms contributing to this observation created a situation where inputs to CA1 were not reliably tuned to incoming spatial information, then it could be possible that in vivo BTSP induction would act on a set of inconsistently active synapses, making any weight modifications difficult to observe. Experiments in the slice controlled for the potential lack of reliable input specificity and allowed appropriate synaptic input-plateau intervals to maximize the temporal overlap between the putative IS and ET. BTSP induction in the slice at the interval most likely to produce maximal potentiation still failed to produce significant increases in synaptic strength in slices from the T286A group but produced a threefold increase in excitatory postsynaptic potential (EPSP) amplitude in slices from WT controls. The combination of in vivo and in vitro experimental results suggests a likely role for αCaMKII outside of the IS or ET mediation, such as synaptic plasticity expression mechanisms. It is possible, however, that the altered CaMKII signaling also affected the interaction of the IS and ET in addition to expression mechanisms, but these alterations could not be detected because of the near complete lack of BTSP.

The spontaneous plateau potentials observed in the T286A animals were longer in duration and occurred more frequently (Fig. 5). A comparison of cellular excitability, measured at the soma, suggested that CA1 pyramidal neurons in the T286A mice had down-regulated excitatory conductances such that AP threshold was more depolarized perhaps in an attempt to compensate for the longer and more frequent plateau potentials. It is known that the probability of plateau potential generation is modulated by entorhinal cortex (EC) input such that silencing direct EC input to CA1 decreases plateau probability (*30*). If the lack of stable PC formation in CA1 associated with the T286A mutation somehow signals the EC to up-regulate activity, then this might contribute to the increase in spontaneous plateaus. In addition, the T286A mutation is believed to down-regulate the activity-dependent activation of dendritic K^+^ currents, specifically those mediating the slow afterhyperpolarization (sAHP) in response to suprathreshold synaptic stimulation (*34*) and inhibiting the autophosphorylation of αCaMKII that blocks the homeostatic up-regulation of hyperpolarization-activated cyclic nucleotide-gated (HCN) channels (*35*). Future investigations will help determine whether the frequency and duration of the spontaneous plateau result from alterations to the network, uncontrolled activity-dependent changes in cellular excitability, or some combination thereof.

Recently, the release of calcium from the endoplasmic reticulum has been demonstrated to be important for regulating behaviorally relevant synaptic plasticity and place field formation in hippocampus (*13*), yet the molecular basis of the ET and IS in BTSP remains elusive. To address this issue, the development of new tools is crucial, particularly those capable of rapidly blocking specific molecules within seconds. Moreover, devising a method for high-throughput screening of BTSP alterations following molecular perturbations would be highly beneficial. Such advancements in technology will not only enhance our comprehension of the BTSP phenomenon but also enrich our knowledge of learning and memory in general. In summary, we have demonstrated an essential role for αCaMKII signaling in BTSP. These results should provide a starting point for future studies to uncover the molecular mechanisms involved in a form of synaptic plasticity known to underlie the formation of PCs and, ultimately, the cellular basis for memory formation in the hippocampus.

## MATERIALS AND METHODS

### Experimental design

We hypothesize that αCaMKII activity might play a role in BTSP. To test this hypothesis, we used αCaMKII T286A mutant mice for BTSP induction experiments. By examining the BTSP induction results, we can determine whether αCaMKII is implicated in BTSP and whether it underlies the ET or the IS or the expression mechanisms.

### Animals and procedures

All experimental procedures were approved by the Baylor College of Medicine Institutional Animal Care and Use Committee (protocol AN-7734). All in vivo experiments were performed as previously described (*8*). In vivo whole-cell recording experiments were performed on 2- to 5-month-old mice of either sex. The αCaMKII T286A mutant mouse line was provided by R. Yasuda [Mouse Genome Informatics ID: 2158733; (*24*)].

Under deep isoflurane anesthesia, custom titanium head bars with an opening above the dorsal hippocampus were affixed to the skull using cyanoacrylate glue and dental cement. Stereotactic coordinates were used to mark the location of the future craniotomy (~0.5 mm) on the skull beneath the head bar, specifically, 1.9 mm posterior from bregma and 1.6 mm lateral from the midline for whole-cell recordings. The recovery period after surgery was a minimum of 7 days. After 2 days of water scheduling, animals underwent 5 days of handling and were acclimated to perform head-fixed on a linear treadmill. Animals were trained to run for a 10% sucrose water reward (~3.5 μl per lap). The training duration for the initial 3 days was 20, 40, and 60 min, respectively. Subsequently, all animals were trained for 1 to 12 days until they reliably completed a minimum of 80 laps per session (60 min). After training, mice were provided with supplemental water if necessary to maintain schedule.

### Linear treadmill

The linear treadmill consisted of a velvet fabric belt with three distinct regions of visual and tactile cues, each region occupying one-third of the belt as previously described (*8*). A custom-made lick port controlled by a solenoid valve (Lee Valve, LHQA1231220H_B) delivered the sucrose water reward. An optical sensor (Panasonic, FX-301H) detected licking. The belt’s location was reset each lap by two photoelectric sensors positioned at the beginning of the linear track. Speed was measured using a rotary encoder affixed to a treadmill wheel axle, and distance was calculated by integrating velocity within a lap. The valve, sensors, and encoder were controlled using a Bpod Finite State Machine (r1.0, Sanworks). A separate custom Arduino-based (Teensy 3.5) module was used to control position-dependent intracellular current injection. All behavioral data were digitized at 20 kHz by a PCIe-6343, X-series DAQ system (National Instruments) using WaveSurfer software (wavesurfer.janelia.org).

### In vivo electrophysiology

Before conducting whole-cell patch recordings, the depth of the CA1 pyramidal layer was ascertained using an extracellular recording electrode. This procedure used a 1.5- to 3-megaohm glass pipette filled with 0.9% NaCl solution. The electrode was positioned vertically on a micromanipulator (Luigs and Neumann), and the extracellular signal was assessed using an audio monitor (A-M systems). The CA1 pyramidal layer was identified by the manifestation of theta-modulated spikes and an increased ripple amplitude. Typically, the depth of the CA1 pyramidal layer ranged from 1000 to 1300 μm below the brain surface. For whole-cell recordings, elongated-taper whole-cell patch electrodes (8 to 12 megaohms) were filled with a solution containing 134 mM potassium gluconate, 6 KCl, 10 Hepes, 4 NaCl, 0.3 Mg–guanosine 5′-triphosphate (GTP), 4 Mg–adenosine 5′-triphosphate (ATP), and 14 tris-phosphocreatine. In some recordings, 0.2% biocytin was added to the intracellular solution. As the patch electrode advanced through the cortex, ~70 kPa positive pressure was applied to avert blockage. At an approximate depth of 100 μm above the pyramidal layer, positive pressure was reduced to ~2 kPa. Upon contact between the electrode tip and a cell, the resistance would increase reproducibly. All neural recordings were performed using a Dagan BVC-700A amplifier in the current-clamp mode and were digitized at 20 kHz by a PCIe-6343, X series DAQ system (National Instruments) using WaveSurfer software (wavesurfer.janelia.org). Bridge balance was adjusted to compensate for series resistance. Recordings with a series resistance exceeding 60 megaohms were excluded from subsequent analyses.

### In vitro electrophysiology

Horizontal hippocampal slices of 400-μm thickness were obtained from 6- to 12-week-old male and female mice using a Leica Vibratome VT1200S. Animals were anesthetized with isoflurane and ketamine/xylazine injection, followed by intracardial perfusion with an ice-cold cutting solution containing 205 mM sucrose, 25 mM NaHCO_3_, 2.5 mM KCl, 1.25 mM NaH_2_PO_4_, 1 mM CaCl_2_, 7 mM MgCl_2_, and 10 mM glucose. The slices were incubated in standard artificial cerebrospinal fluid (aCSF) (below) for 40 min at 35°C before being maintained at room temperature. Whole-cell current-clamp recordings were conducted at 33° to 34°C in aCSF perfused into a submerged recording chamber at a rate of 2 ml/min. The aCSF composition varied depending on the experiment and contained the following: 125 mM NaCl, 25 mM NaHCO_3_, 2.5 mM KCl, 1.25 mM NaH_2_PO_4_, 2 mM CaCl_2_, 1 mM MgCl_2_, and 16 mM glucose (for BTSP experiments); and 125 mM NaCl, 25 mM NaHCO_3_, 3 mM KCl, 1.25 mM NaH_2_PO_4_, 1.3 mM CaCl_2_, 1 mM MgCl_2_, and 16 mMglucose (for all other experiments). All aCSF solutions contained fresh 3 mM sodium pyruvate and 1 mM ascorbic acid and were constantly bubbled with 95% O_2_ and 5% CO_2_.

Cells were visualized using a Zeiss Examiner Z1 microscope with a water-immersion lens (63×, 1.0 numerical aperture, ZEISS) using Dodt contrast. Whole-cell patch recordings were performed using a Dagan BVC-700 in current-clamp mode, analog-filtered at 1 kHz, and digitized at 50 kHz using a PCIe-6343, X series DAQ system (National Instruments), controlled by Neuromatic software (*36*). Patch electrodes (5 to 7 megaohms) were filled with filtered internal solutions, varying based on the experiment. For BTSP experiments, the solution contained the following: 130 mM Cs-methanesulfonate, 6 mM KCl, 10 mM Hepes, 4 mM NaCl, 4 mM Mg-ATP, 0.3 mM tris-GTP, and 14 mM tris-phosphocreatine. For all other experiments, the solution contained the following: 134 mM potassium gluconate, 6 mM KCl, 10 mM Hepes, 4 mM NaCl, 0.3 mM Mg-GTP, 4 mM Mg-ATP, 14 mM tris-phosphocreatine, and 0.2% biocytin.

Series resistance was maintained between 18 and 45 megaohms when using Cs^+^ to optimize cell health as previously described (*8*). EPSPs were induced by extracellular stimulation (0.1 ms, 0.01 to 0.05 mA) of axons in the *stratum radiatum* region using a platinum-iridium microelectrode (0.5 megaohms, World Precision Instruments) positioned 100 to 300 μm from the recorded cell. Input resistance was monitored using a 150-ms and −30-pA current injection following the EPSP test stimulation. For BTSP experiments, gabazine (SR 95531, 2 μM) and CGP 55845 (50 nM) were added to the aCSF solution, and the CA3 region was removed to prevent epileptiform activity.

The induction of BTSP was performed as described previously (*8*). Briefly, the synaptic inputs and the plateau potential were paired such that the midpoint of the 20-Hz train was coincident with the onset of current injection used to elicit the plateau potential (300 ms, 300 to 600 pA). This 0-ms time interval was chosen because it produces maximal potentiation (*8*). We used five pairings with a 20-s interval. This interval is similar to the time required for animals to complete one lap in our in vivo recordings.

### Cell inclusion criteria for in vivo recordings

A subset of cells was included for analysis (fig. S4), based on the following criteria: (i) The cell’s after-hyperpolarization (AHP) was quantified by determining the mode of all AP AHPs, with the requirement that it should exceed −5 mV. (ii) The mean square error of the membrane potential (*V*_m_) distribution fit to a Gaussian function should be more than 4.5 × 10^−6^. Criteria (i) and (ii) were designed to identify potential interneurons (*33*). (iii) The animal’s locomotion during the induction laps should not include more than 10 stops (excluding stops at the reward site to consume the reward). (iv) The licking probability for areas outside the reward zone should be less than 0.1. The reward zone was defined as 12.6 cm before the reward and 14.4 cm after the reward. Criteria (iii) and (iv) aimed to exclude cells correlated with poor animal behavior. (v) The AP amplitude should be greater than 35 mV relative to threshold. The AP amplitude is indicative of recording quality, with higher AP amplitudes signifying better recording quality. This criterion was established to exclude cells with poor recording quality. (vi) The most hyperpolarized part of the ramp (five-bin average) should not exceed 8 mV. This criterion was set to exclude cells in which the BTSP induction was too close to the initiation of the recording when cells are transiently hyperpolarized. (vii) Cells from the CA3 region were also excluded. As a result of the selection process, 88 of the 125 recorded cells (70.4%) were included. However, quantification of the entire 125 cells revealed similar results (both the behavior and the BTSP) to the selected 88 cells (figs. S4 and S5, A to D), suggesting that the cell selection process did not affect the final conclusions.

### The correlation of behavior and BTSP

Animal behavior quantified as licking and running was not significantly correlated with the BTSP ramp formation (fig. S5, E and F), suggesting that the difference in the behavior of CaMKII homozygous mutant group does not affect the BTSP induction experiment.

### Data analysis

For the analysis of the ramp of depolarization, APs were eliminated by removing all data points 1.5 ms before and 3.5 ms after a designated threshold value (the maximum second derivative with respect to voltage) and low-pass–filtered (<3 Hz) as described previously (*8*). The subthreshold membrane potential was spatially binned into 100 bins, each approximately 1.8 cm in size. The average *V*_m_ from five laps preceding BTSP induction and five laps following the induction was calculated to determine the before trace and after trace. The ramp was computed by subtracting the before trace from the after trace, subsequently converting it to the time domain based on the fastest running speed during the five induction laps. To quantify the peak of the ramp, the membrane potential from 1 s before running and 1 s after the animal completed running was incorporated into the ramp (with 20 time bins added before the 100 spatial bins and 20 time bins after the spatial bins). The average *V*_m_ recorded in the 20 time bins preceding the animal’s initiation of movement served as the baseline for the ramp and was subtracted from all values to set the baseline *V*_m_ to 0 mV. The ramp peak was defined as the maximum value of the baseline-subtracted subthreshold *V*_m_, and the ramp’s area was calculated from the integral of the subthreshold *V*_m_.

Spike frequency accommodation was analyzed by examining AP trains resulting from a 350-pA current injection, as the number of APs in the WT and Homo groups was not statistically different at this current injection intensity (Fig. 6F). Spike accommodation was quantified by dividing the first interspike interval by the last interspike interval.

Animal behavior was analyzed by quantifying the animal’s licking behavior and running speed. The animal’s running speed was calculated using the average of all the recorded laps of the spatially binned running speed. Grouped data shown in Fig. 2E are the mean and SEM of each recorded cells in three groups. The spatial bin that has the minimum average running speed was used for comparison between the three groups as shown in Fig. 2F. The licking probability was calculated for each spatial bin, with a probability of 1 if a lick was detected and 0 if no lick was detected. The relative licking probability increase in the reward zone was calculated as the averaged licking probability within the reward zone minus the averaged licking outside the reward zone, divided by the averaged licking outside the reward zone, as defined by the following equation

$$\text{licking increase rate}=\frac{P_{\mathrm{licking}\;\mathrm{in}\;\mathrm{reward}\;\mathrm{zone}}-P_{\mathrm{licking}\;\mathrm{outside}\;\mathrm{reward}\;\mathrm{zone}}}{P_{\mathrm{licking}\;\mathrm{outside}\;\mathrm{reward}\;\mathrm{zone}}}$$

The reward zone was defined as 12.6 cm before and 14.4 cm after the reward. This method was used for quantifying licking behavior. Ideal licking behavior should exhibit high licking probability within the reward zone and very low licking probability outside of the reward zone.

### Statistical methods

Sample sizes were not predetermined using statistical methods. Data analysis was performed using two-sample Student’s *t* test or one-way analysis of variance (ANOVA) with Bonferroni correction, as indicated in the figure legends. Nonparametric tests used the Kruskal-Wallis test with Dunn’s test for multiple comparisons. The normality of the data was assessed using the Shapiro-Wilk parametric hypothesis test, using a MATLAB script (Ahmed BenSaïda, www.mathworks.com/matlabcentral/fileexchange/13964-shapiro-wilk-and-shapiro-francia-normality-tests) and by visually examining the quantile-quantile plot of the data. For datasets with large sample sizes (Fig. 5B), a permutation test with Bonferroni correction was conducted to avoid type I errors. The permutation test was performed using a MATLAB script (Laurens R Krol, https://github.com/lrkrol/permutationTest), master branch, updated on 13 January 2021.
