# Supplementary material for: A critical role for CaMKII in behavioral timescale synaptic plasticity in hippocampal CA1 pyramidal neurons
Source: Sci Adv. 2023 Sep 6;9(36):eadi3088. doi: 10.1126/sciadv.adi3088 (PMC10482326; doi:10.1126/sciadv.adi3088)
Supplement: Supplementary file 1 — Figs. S1 to S5 [file sciadv.adi3088_sm.pdf]

Science Advances
AAAS

Supplementary Materials for

A critical role for CaMKII in behavioral timescale synaptic plasticity in hippocampal CA1 pyramidal neurons

Kuo Xiao *et al.*

Corresponding author: Jeffrey C. Magee, jeffrey.magee@bcm.edu

Sci. Adv. **9**, eadi3088 (2023)
DOI: 10.1126/sciadv.adi3088

This PDF file includes:

Figs. S1 to S5

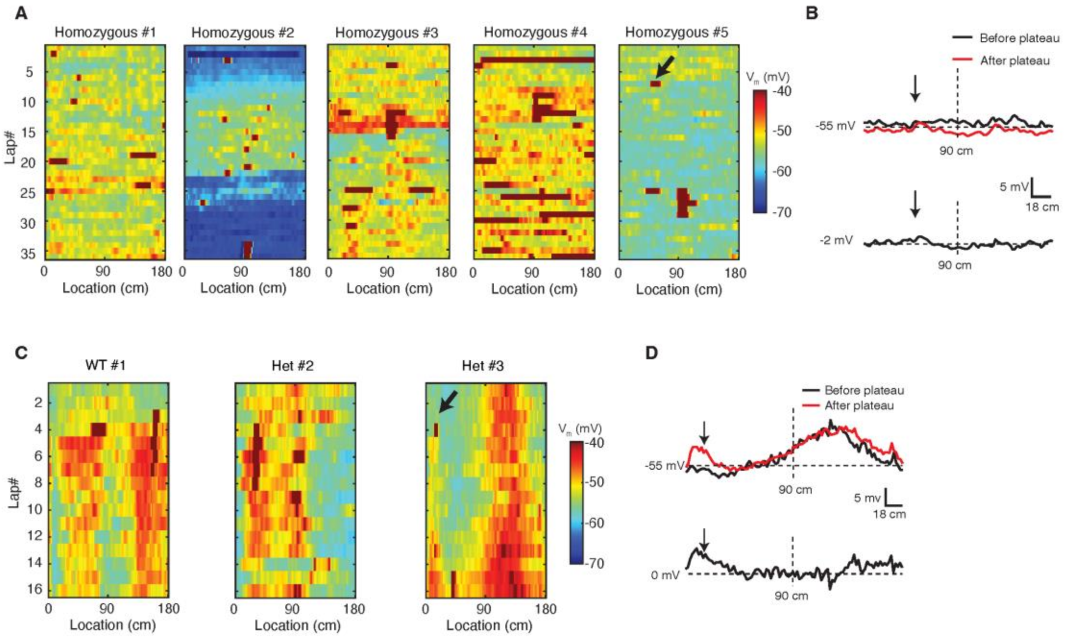

A
Homozygous #1
Homozygous #2
Homozygous #3
Homozygous #4
Homozygous #5
Lap#
Location (cm)
V_m (mV)
B
Before plateau
After plateau
-55 mV
90 cm
5 mV
18 cm
-2 mV
C
WT #1
Het #2
Het #3
D
0 mV
5 mv

Fig. S1.

Spontaneous plateau potentials do not induce place field formation in the CaMKII homozygous mutant mice

(A), 5 example cells show the spontaneous plateau potentials in the CaMKII homozygous mutant mice do not induce place field formation. Note that the black arrow indicates a spontaneous plateau event. (**B)**, Plot of the subthreshold membrane potential before and after the spontaneous plateau event. This Plot corresponds to the plateau event marked by the black arrow in panel A. Plateau location is marked on top of the traces by arrowheads. (**C)**, 3 examples of spontaneous plateaus in both the WT and the Het group. (**D)**, Plot of the subthreshold membrane potential before and after the spontaneous plateau event. This Plot corresponds to the plateau event marked by the black arrow in panel C. Plateau location is marked on top of the traces by arrowheads.

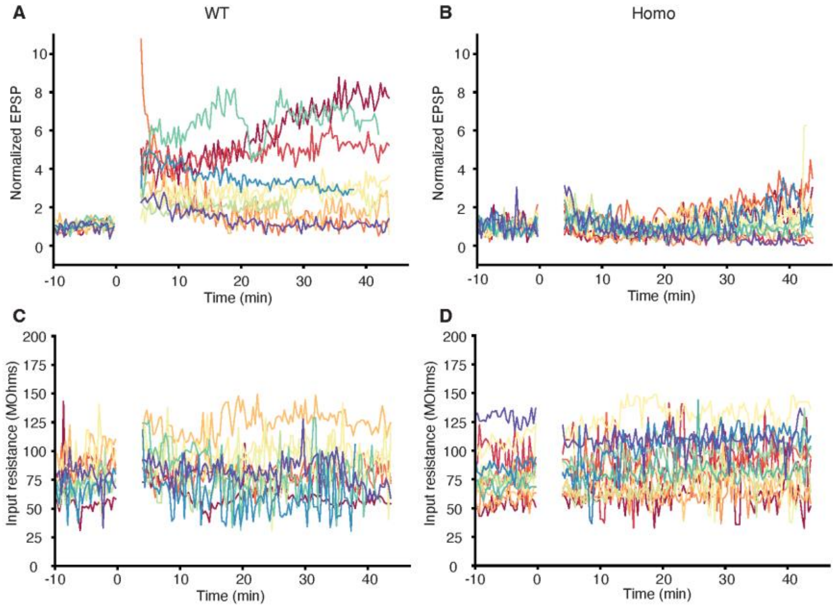
A
WT
B
Homo
Normalized EPSP
Time (min)
C
D
Input resistance (MOhms)
Time (min)

Fig. S2.

All neurons tested for BTSP in slice

(A), BTSP induction causes EPSP amplitude to increase in cells from WT group. (**B)**, BTSP induction is less effective in the cells from Homo group. (**C-D**), The input resistance was monitored during the recording. n=10 from 8 mice for WT, n=12 from 5 mice for Homo.

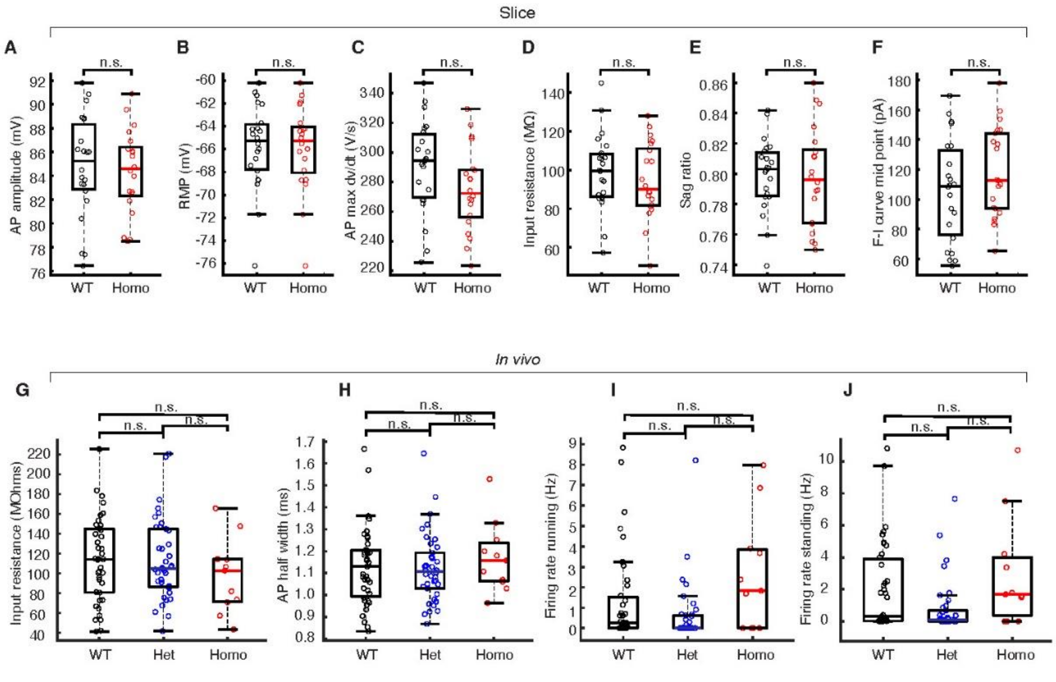

Slice
A
B
C
D
E
F
n.s.
AP amplitude (mV)
RMP (mV)
AP max dv/dt (V/s)
Input resistance (MΩ)
Sag ratio
F-I curve mid point (pA)
WT
Homo
In vivo
G
H
I
J
Input resistance (MOhms)
AP half width (ms)
Firing rate running (Hz)
Firing rate standing (Hz)
WT
Het
Homo

Fig. S3.

A comparison of membrane properties between the CaMKII T286A and WT neurons
(A), quantification of the action potential amplitude in WT and Homo groups cells recorded from brain slices. T statistic: 0.370, P-value: 0.713. (**B)**, quantification of the resting potential in WT and Homo groups cells recorded from brain slices. T statistic: 0.133, P-value: 0.895. (**C)**, quantification of the max dv/dt in action potential in WT and Homo groups cells recorded from brain slices. T statistic: 1.73, P-value: 0.0912. (**D)**, quantification of the input resistance in WT and Homo groups cells recorded from brain slices. T statistic: 0.748, P-value: 0.458. (**E)**, quantification of the sag ratio in WT and Homo groups cells recorded from brain slices. T statistic: –0.203, P-value: 0.840. (**F)**, quantification of the mid-point in the F-I curve fitted to a sigmoid function. T statistic: –1.23, P-value: 0.222. (**G)**, comparison of input resistance recorded in-vivo in WT, Het and Homo groups. F statistic: 0.7, P-value: 0.499 for the ANOVA test. T statistic: –0.0547 (WT vs. Het), 1.09 (WT vs. Homo), 1.16 (Het vs. Homo). P-value: 1.00 for WT and Het; 0.825 for WT and Homo; 0.782 for Het and Homo. (**H)**, comparison of action potential half width recorded in-vivo in WT, Het and Homo groups. H statistic: 0.77, P-value: 0.68 for the Kruskal-Wallis test. Z statistic: 0.178 (WT vs. Het), –0.656 (WT vs. Homo), –0.922 (Het vs. Homo). P-value: 0.995 for WT and Het; 0.845 for WT and Homo; 0.765 for Het and Homo. (**I)**, quantification of the standing firing rate of 5 laps before BTSP induction. H statistic: 6.26, P-value: 0.0438 for the Kruskal-Wallis test. Z statistic: 2.02 (WT vs. Het), –0.459 (WT vs. Homo), –2.14 (Het vs. Homo). P-value: 0.106 for WT and Het; 0.903 for WT and Homo; 0.130 for Het and Homo. (**J)**, quantification of the running firing rate of 5 laps before BTSP induction. H statistic: 5.19, P-value: 0.0746 for the Kruskal-Wallis test. Z statistic: 1.64 (WT vs. Het), –1.08 (WT vs. Homo), –1.97 (Het vs. Homo). P-value: 0.294 for WT and Het; 0.679 for WT and Homo; 0.112 for Het and Homo. Two sample student's t-test was conducted in all slice experiment analysis. One-way ANOVA with Bonferroni correction was conducted in all parametric *in-vivo* experiment statistical analysis. Kruskal Wallis test with Dunn's test for multiple comparisons in non-parametric analysis. *P< 0.05, **P< 0.01 and ***P< 0.001; NS, not significant (P≥ 0.05). Slice experiments sample sizes: n=22 (WT) from 3 mice, n=20 (Homo) from 3 mice. In-vivo sample sizes: n=39 (WT) from 24 mice, n=38 (Het) from 26 mice, n=11 (Homo) from 7 mice.

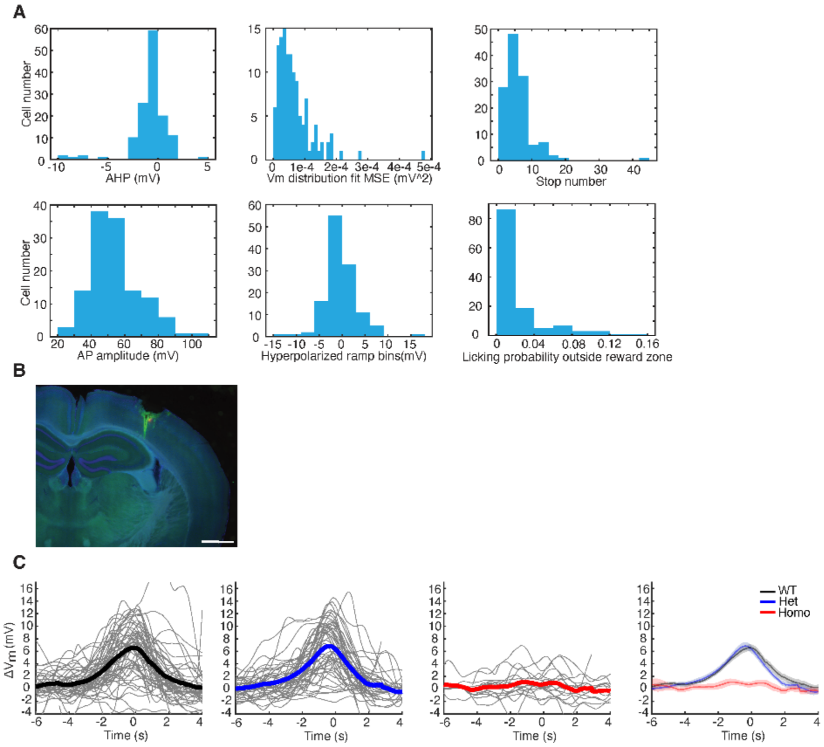

Fig. S4.

Cell selection does not affect BTSP ramp analysis results

(A), The distribution of 6 parameters that are used in cell selection criteria (see Methods). Cell selection criteria contains 7 standards: 1. The cell's AHP (mode of all action potential's AHP) should be > –5 mV. 2. The mean squared error of V_m distribution fit to gaussian function should be more than 4.5e–6. The purpose for standards 1 and 2 is to exclude potential interneurons (see (34)). 3. The animal's average stop number during the induction period should be less than 10 times (excluding the stop at the reward site to consume the reward). 4. The licking probability for outside reward zone should be less than 0.1. reward zone is defined as 12.6 cm before reward and 14.4 cm after reward. 5. The AP amplitude should be > 35 mV. In *in-vivo* recordings, the AP amplitude is a good indication of the recording quality, with higher AP amplitude, the recording quality is better. Here the standard is to exclude cells that has poor recording quality. 6. The most hyperpolarized part of ramp (5 bins average) should not be > 8 mV. This standard is to exclude cells that have BTSP induction too close to the beginning of the recording. 7. Cells from CA3, see panel B. (**B)**, example of cell from CA3 region. scale bar, 1 mm. **C**, plot of ramps from all cells collected in WT Het and Homo groups. Note that the shape of the ramp is very similar to the cells selected for analysis.

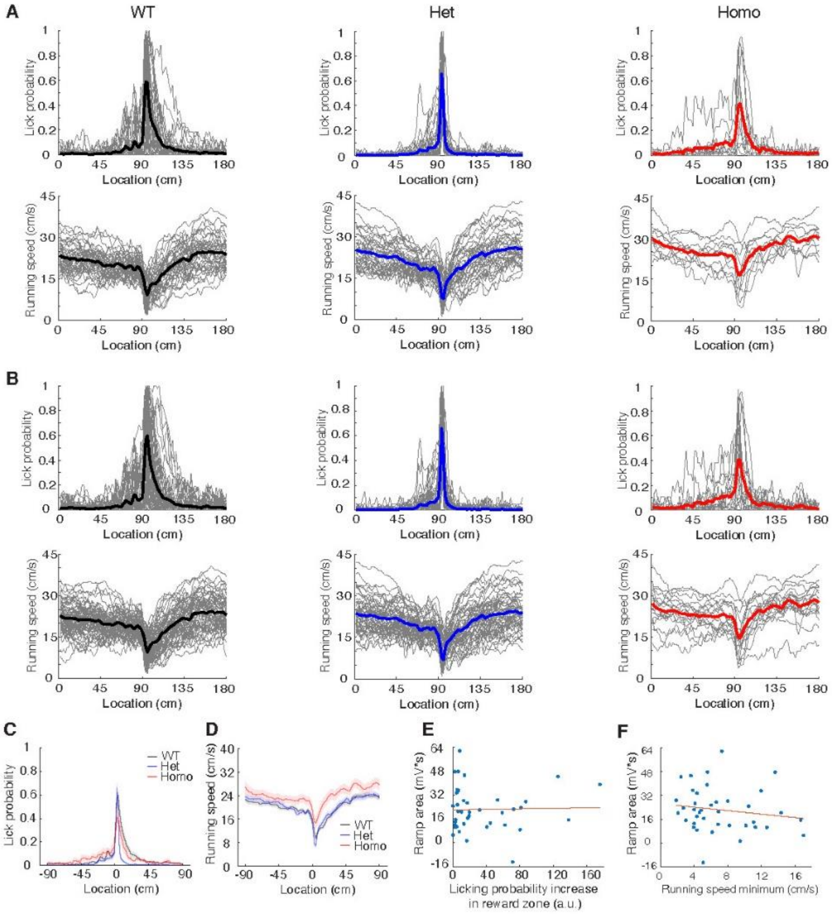
A
WT
Het
Homo
Lick probability
Location (cm)
Running speed (cm/s)
B
C
D
E
F
WT
Het
Homo
Ramp area (mV*s)
Licking probability increase
in reward zone (a.u.)
Running speed minimum (cm/s)

Fig. S5.

Cell selection does not affect the behavior analysis results

(A), The licking and running traces from the subset of cells that have been selected using the inclusion criteria (N=88, see methods). (**B)**, The licking and running traces from all cells (N=125) that have been recorded. (**C)**, The averaged traces of the licking probabilities in WT, Het and Homo groups from all cells recorded. The central line and shaded background represents mean ± SEM, respectively. (**D)**, The averaged traces of the running speeds in WT, Het and Homo groups from all cells recorded. (**E)**, The correlation between the animal behavior (licking) and the BTSP ramp. The regression showed that the Licking was not (or very weakly) correlated with the BTSP ramp. R-square for regression: 5.67e–4. (**F)**, The correlation between the animal behavior (running) and the BTSP ramp. The plot showed the BTSP ramp area plotted against the minimum running speed. R-square for regression: 0.0260. n=58 for WT, n=50 for Het, n=17 for Homo in all cells. N=39 for WT, n=38 for Het, n=11 for Homo in selected cells.
